# Six Sigma can significantly reduce costs of poor quality of the surgical instruments sterilization process and improve surgeon and operating room personnel satisfaction

**DOI:** 10.1038/s41598-023-41393-x

**Published:** 2023-08-29

**Authors:** Andrea Saporito, Claudio Tassone, Antonio Di Iorio, Marcella Barbieri Saraceno, Alessandro Bressan, Ramon Pini, Francesco Mongelli, Davide La Regina

**Affiliations:** 1 Department of Anesthesia, Ospedale Regionale di Bellinzona e Valli, EOC, Bellinzona, Switzerland; 2 Faculty of Medicine, Università della Svizzera Italiana, Lugano, Switzerland; 3 Operating Theatre, Ospedale Regionale di Bellinzona e Valli, EOC, Bellinzona, Switzerland; 4 Hospital Direction, Ospedale Regionale di Bellinzona e Valli, EOC, Bellinzona, Switzerland; 5 Department of Surgery, Ospedale Regionale di Bellinzona e Valli, EOC, Via Gallino 12, 6500 Bellinzona, Switzerland

**Keywords:** Health care economics, Surgery

## Abstract

Operating room (OR) management is a complex multidimensional activity combining clinical and managerial aspects. This longitudinal observational study aimed to assess the impact of Six-Sigma methodology to optimize surgical instrument sterilization processes. The project was conducted at the operating theatre of our tertiary regional hospital during the period from July 2021 to December 2022. The project was based on the surgical instrument supply chain analysis. We applied the Six Sigma lean methodology by conducting workshops and practical exercises and by improving the surgical instrument process chain, as well as checking stakeholders’ satisfaction. The primary outcome was the analysis of Sigma improvement. Through this supply chain passed 314,552 instruments in 2022 and 22 OR processes were regularly assessed. The initial Sigma value was 4.79 ± 1.02σ, and the final one was 5.04 ± 0.85σ (SMD 0.60, 95%CI 0.16–1.04, p = 0.010). The observed improvement was estimated in approximately $19,729 of cost savings. Regarding personnel satisfaction, 150 questionnaires were answered, and the overall score improved from 6.6 ± 2.2 pts to 7.0 ± 1.9 pts (p = 0.013). In our experience the application of the Lean Six Sigma methodology to the process of handling the surgical instruments from/to the OR was cost-effective, significantly decreased the costs of poor quality and increased internal stakeholder satisfaction.

## Introduction

Six Sigma is a methodology for process continuous improvement born in the 1980s at Motorola and subsequently successfully applied to different processes^1^, in different contexts, and by many manufacturing companies^2^. It consists of the systematic application of problem-solving techniques, consequent structured implementation of improvements, and the use of process behavioral studies to maintain the achievements^3^. It implies the systematic collection and statistical analysis of data to understand a given, potential process optimization margin and subsequent reorganization of that process to improve it, thus meeting customers’ expectations. In the context of modern complexity, the ‘customer’ is any stakeholder in the process, from the next person who utilizes the intermediate output of a micro-process within the organization (internal customer), to the end-user of the finished product (external customer)^4,5^. This interpretation of the concept of pervasive customer satisfaction, functional to the achievement of the so-called ‘total quality’ within the organization, is central in the Six Sigma philosophy and is reflected by its methodology, based on the SIPOC identification, i.e. the clear identification, within each micro-process by which the process is composed, of Suppliers, Inputs, Process, Outputs, and Customers^6,7^.

One of the fundamental assumptions of the whole theoretical complex of this methodology is the interpretation of the process variability as the main source of errors, inefficiency, and poor quality. The variability shall thus be reduced as much as possible to eliminate the risk of defects in the final product or service and to make it a reliable, safe, and capable process. To understand a process performance with specific regard to its fallacy, the analysis of the mean is often useless. A mean per se may be in line with the industry standards, while a process can still contain plenty of defects (deviation from the standard). Those defects do not refer uniquely to the final product, as they may occur at different levels and within each micro-process, by which the process is composed^8^. To understand the performance of a given process, however, the absolute total number of its measured defects shall be related to the total number of defects opportunities. The latter is, in more prosaic words, the sum of all the different ways something can go wrong, at all levels, across the whole process. A normal process can be composed by tens or hundreds of micro-processes, each with a SIPOC structure, which may at some point diverge from their gold standard in one or more given aspects, possibly accounting in turn for thousands of different errors possibilities, defined as *opportunities* of the defect^9,10^.

**Figure 1:**
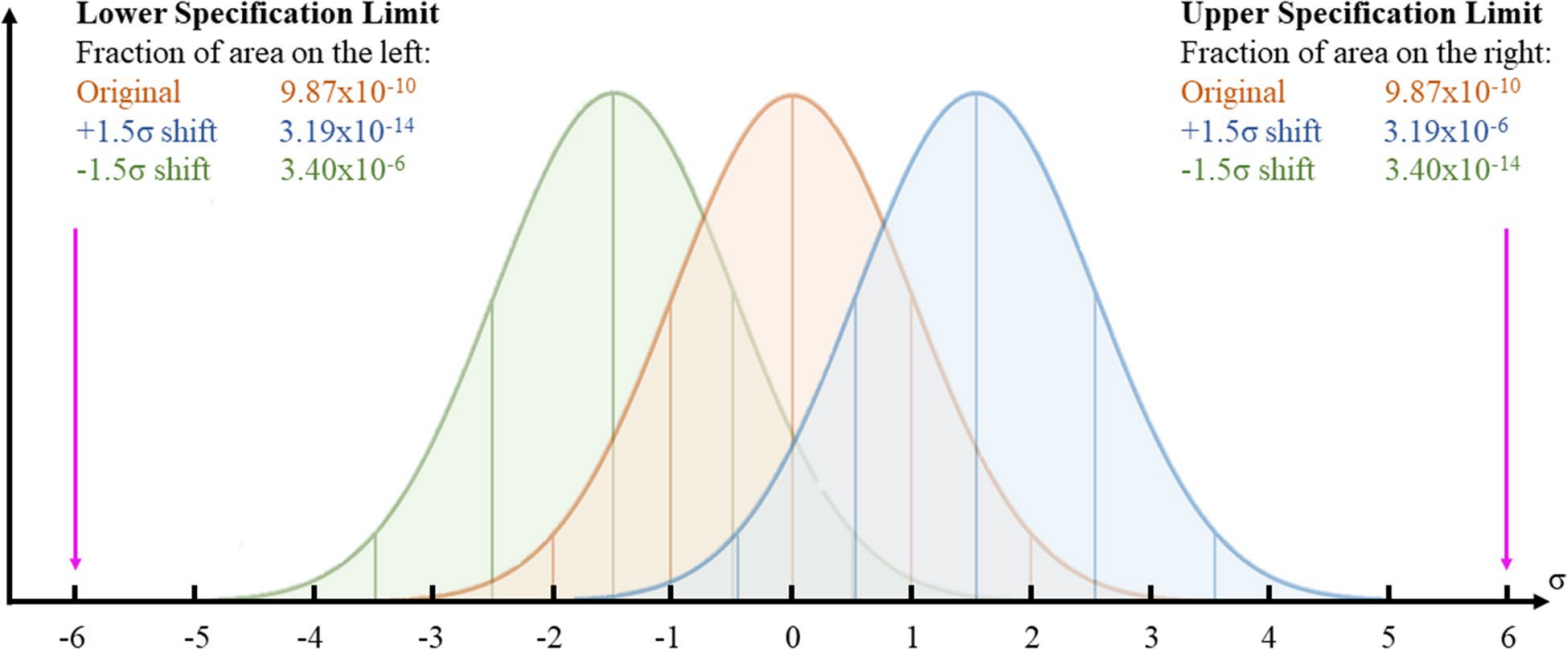
Normal distribution. The horizontal axis showed the distance from the mean, denoted in units of standard deviation (represented as σ). The greater the standard deviation, the larger the spread of values. The upper and lower specification limits (USL and LSL) are at a distance of 6 σ from the mean.

The Six Sigma method interprets quality in terms of measured defects per million opportunities, defining the standard of excellence as a process with no more than 3.4 defects per million opportunities (DPMO) and aiming, by the systematic application of its different tools and the Define, Measure, Analyze, Improve, and Control (DMAIC) approach, at meeting this standard. The very name *Six Sigma* refers precisely to this statistical concept, as a value of 3.4 DPMO falls within the interval defined by six units of standard deviation (σ) in a normal distribution, whereas the greater the standard deviation from the mean (µ), the larger the spread of values (i.e. the variability from a standard represented by the mean itself). Figure 1. Table 1 visualizes the exponential variation of the DPMO value and percent of defects with the σ value: the reduction of 1 σ corresponds to a radical mitigation of the risk of error^11^.

**Table 1:** Variation of defects per million opportunities (DPMO) and defects with σ (sigma) levels.

Sigma level	Sigma	DPMO	Percent defective
1	− 0.5	691,462	69%
2	0.5	308,538	31%
3	1.5	66,807	6.7%
4	2.5	6210	0.62%
5	3.5	233	0.023%
**6**	**4.5**	**3.4**	**0.00034%**
7	5.5	0.019	0.0000019%

A level of 6 σ corresponds to 3.4 DPMO (marked in bold).

Although the main aim of Six Sigma is achieving the highest quality standards possible, this also has a strong economic implication, as defects invariably lead to a loss in cost-effectiveness. The direct and indirect –often hidden- costs associated with defects within a process are defined as costs of poor quality (COPQ). The reduction of COPQ and the related increase in the process overall cost-effectiveness is a consequence of the reduction in the process variation.

Given those premises, during the last two decades, Six Sigma has been successfully and progressively adopted by the tertiary (service) sector as well. Its application in the industrial sector is marked by its rigorous approach to process improvement and quality enhancement. By meticulously analyzing processes and minimizing variations, it leads to tangible benefits. For instance, in a manufacturing setting, Six Sigma can identify and rectify the root causes of defects in product components. This not only improves the final product quality but also reduces the need for rework, resulting in cost savings and increased customer satisfaction^12^. In the aerospace industry, Six Sigma can optimize complex assembly processes. By analyzing data and identifying bottlenecks, the methodology enhances production efficiency and reduces errors. This can lead to reduced lead times in aircraft manufacturing, translating to significant financial gains and improved delivery schedules. In electronics manufacturing, Six Sigma aids in identifying sources of variation that lead to inconsistent product performance. By implementing solutions derived from statistical analysis, the sector can produce more reliable and consistent electronic components^13^.

Six Sigma methodology has been also applied in the healthcare sector. In a progressive trend toward healthcare industrialization and under extreme cost pressure in Switzerland, Six Sigma has been increasingly incorporating in diverse aspects of the healthcare sector during the last years. The methodology is lending itself to its high-reliability requirements and zero tolerance for mistakes. Some of the aspects where Six Sigma has been proven effective in reducing defects in healthcare processes are inventory optimization, care delivery, and administrative processes efficiency^14^. Six Sigma appears to be most effectively applied in fields that share similarities with the manufacturing industry, where high volumes are produced daily, and processes can be easily standardized. The healthcare sector and manufacturing industry share several similarities when it comes to achieving efficient production processes, despite their distinct natures^15^. Both sectors aim to optimize processes to minimize waste, enhance efficiency, and improve outcomes. In healthcare, process optimization includes enhancing patient flow, reducing waiting times, and optimizing resource utilization. Moreover, both sectors prioritize quality control to ensure consistent and reliable outcomes, increasingly rely on data-driven decision-making, and aim at standardizing processes such as protocols and guidelines to ensure uniformity in treatments and patient care. Both apply the lean methodology that focuses on waste reduction and continuous improvement, and a customer-centric approach. Both industries prioritize delivering value to their end users. In manufacturing, this involves meeting customer requirements and expectations for product performance. In healthcare, the focus is on patient-centered care, tailoring treatments to individual needs. While healthcare and manufacturing differ in their products and services, they both strive for efficient production processes that result in high-quality outcomes. The mentioned shared principles underpin their pursuit of efficiency^12,13^.

Operating room (OR) management is a complex and multidimensional managerial activity, which combines many aspects of care. Besides the clinical ones, many processes deal with the maximization of the cost-effectiveness of the service^16,17^. The latter can be achieved mainly via a reduction of both the underutilized operating room time and the overruns, a maximization of the time dedicated to value-added activities, and a reduction in COPQ. An operating suite has many internal and external stakeholders that significantly impact its efficiency. One of the most central and delicate process is the sterilization of surgical instruments, which presents several similarities with a standard manufacturing process^18^. Both prioritize standardized processes, quality control, resource optimization, data management, risk mitigation, Lean principles, supply chain management, workforce training, and regulatory adherence to ensure safe and consistent outcomes in their respective domains. Such domains are of utmost importance in OR for ensuring timely availability of medicines, medical equipment, and other critical resources while ensuring efficiency and patient safety^19,20^.

Despite the substantial success of Six Sigma in various industries, its application within the healthcare sector remains underexplored. A research gap exists in understanding how Six Sigma principles can be tailored to address the unique challenges of healthcare processes, such as patient safety, complex workflows, and regulatory compliance. There is limited empirical research that specifically focuses on implementing and evaluating the impact of Six Sigma in healthcare settings. Our research explored how Six Sigma principles, widely adopted in manufacturing and other industries, can be effectively adapted to healthcare processes. This longitudinal observational study aims to assess the impact over time of the application of the Six Sigma methodology for the optimization of the process of surgical instruments sterilization in a public general hospital with a centralized sterilization service.

## Materials and methods

The requirement for informed consent from the study subjects and protocol approval were waived by the local ethic committee (Comitato Etico Cantonale Ticino) due to the retrospective study design and as patient data was fully anonymized. The present research was performed in accordance with relevant national guidelines/regulations and with the Declaration of Helsinki.

### Setting and objectives

The project was conducted at the operating theatre with 5 operating rooms of the Bellinzona and Valli Regional Hospital, Bellinzona, Switzerland in collaboration with the central sterilization center of the Ente Ospedaliero Cantonale (EOC) located approximately 30 km away from the hospital during the period July 2021 to December 2022. All EOC operating rooms of our hospital network refer to the centralized sterilization center. For this study, we analyzed only the OR of the Bellinzona e Valli Regional Hospital.

The project was based on the analysis of the surgical instrument supply chain; from the used instrument after the surgical interventions, to the shipment to the sterilization center and reprocessing with subsequent resterilization, concluding with the shipment back to the hospital and its storage of the ready to be used instruments until the next scheduled surgical intervention. Such a process consists of up to 58 different, subsequent steps and hand-overs leading to the creation of various types of errors (delays, additional rework costs, use of highly paid taxis for urgent transportation, damage, etc.).

The primary outcome was the reduction in defects per million opportunities (DPMO); secondary outcomes were the reduction of related and the increase in the main stakeholders’ (surgeons and OR personnel) satisfaction, measured as Net Promoter Score (NPS).

### Design

This research project was chosen as part of a cyclic and interdisciplinary collaboration process in which team members worked by applying the DMAIC logic through the stages described in Deming's cycle (Plan-Do-Check-Act), which are foundational tools of lean thinking. In addition, we applied the Six Sigma methodology through the application of statistical analysis and monitoring of DPMO^21^.

The project was initiated by a multidisciplinary team led by the anesthesia department head and medical director of the operating block, and the nursing manager of the operating block, whom both decided to deepen their knowledge by pursuing Lean Management and Six Sigma concepts. This was done with the support and guidance of the director of the Regional Hospital of Bellinzona and Valli, who has extensive experience in lean methodology and transformation programs and developed over 25 years of prior service in the industrial sector. Subsequently, a team consisting of three operating room technicians and four care assistants was integrated into the project's data collection and subsequent re-engineering phases.

The project took place over 2 years, from January 2021 to December 2022, and is still ongoing. For this paper, the period from July 2021, the month in which data collection officially began after the preparatory phase, to December 2022 was analyzed.

In the first 6 months of the project, workshops were initially conducted, led by a Lean Six Sigma master, as a theoretical approach to the methodology and practical exercises aimed at understanding and executing the fundamentals. Subsequently, work began on constructing the initial snapshot (process As-Is) of the surgical instrument process chain. Project objectives were:

1. Standardize the process to improve reliability and capability
2. Reduce process variations
3. Improve the cost-effectiveness of the process
4. Eliminate waste or non-value-added activities such as reduction of the number of errors/reworks
5. Increase stakeholder satisfaction
6. Reach a predictable level of quality
7. Create an efficient continuous flow

We first started by mapping the current state (process As-Is) of the surgical instrument supply chain, allowing for a detailed description of the process analyzed to identify critical points, value-adding (VA) activities, and non-value-adding activities. VA activities were defined as those actions improving the safety of patients, service accessibility, sustainability (cost-effectiveness ratio), working conditions for surgeons and healthcare professionals (perceived quality), reproducibility, and decreasing waiting times and waste. The COPQ was defined as costs attributable to improvable performances in the processes, which are generally sustained by process defects or as a consequence of system defects^4^. Table 2. By applying this methodology, it was possible to identify non-value-adding activities, divided into direct ones for the hospital and indirect ones for the sterilization center.

**Table 2:** Costs of poor quality.

Costs of poor quality	Controllable poor-quality cost	Prevention cost	Quality planning (for test, inspections, audits, process control) Education and training Performing capability analyses Conducting design reviews
		Appraisal cost	Test and inspection Supplier acceptance sampling Auditing processes
	Resultant poor-quality cost	Internal error cost	In-process scrap and rework Troubleshooting and repairing Design changes Additional inventory required to support poor process yields and rejected lots Reinspection and retest of reworked items Downgrading
		External error cost	Sales returns and allowances Service level agreement penalties Complaint handling Field service labor and parts costs incurred due to warranty obligations

To further identify processes to improve, we applied the SIPOC diagram (Supplier, Input, Process, Output, Customer), which allowed us to construct a macroscopic current state map of the process under examination. To construct the diagram according to the points defined by the acronym, the steps to be taken were:

1. Identify both direct and indirect customers
2. Identify the process outputs (operating instructions, protocols)
3. Identify the macro-process phases (start and end points of the process)
4. Determine the Process Owner
5. Identify the process inputs: what is used in the process, tools, equipment, personnel

Starting from the macro-process, we applied a top-down approach to analyzing in detail up to the desired level of depth, the so-called point-of-impact. We also could distinguish between value-adding and non-value-adding activities. The SIPOC diagram should be filled out starting from the end of the process (Customer) and working backward to the beginning of the process (Supplier)^11^. By following the above-described steps and applying them to the context at hand, it was possible to construct a SIPOC starting from the Process map.

In our study, we also collected NPS as a parameter for objectifying the degree of satisfaction and approval of the personnel. It was administered not only to internal stakeholders, namely surgeons, but also to the staff working in the process chain, constituted by operating room technicians, instrument nurses, and auxiliary care personnel. We distinguished among three different values that were collected for each category and thus understanding the level of satisfaction in the chain as well as the commitment of the workers. We evaluated the satisfaction of surgeons, support equipe and OR nurses with a structured questionnaire reporting the following:

- Overall, how likely would you recommend other surgeons to operate with us?
- How satisfied are you with the organization of the surgical instruments overall?
- How often do defects occur in the instruments?
- How often do you have to wait to schedule a surgery due to the lack of availability of the instruments?
- How satisfied are you with the support provided by the instrument staff during the surgery?
- How safe do you feel working with the instruments at your disposal?
- How satisfied are you with the overall organization of the operating room?

The questionnaire was systematically administered during the first and the last 6 months and for each question a score from 0 to 10 was attributed.

### Statistical analysis

Descriptive statistics were presented as absolute frequencies for categorical variables and mean with standard deviation (SD) for continuous variables. For the comparison of continuous variables, the Student-t test for paired values has been used. For comparisons of interest also standardized mean difference (SMD) with a 95% confidence interval (95%CI) was provided. Kaplan–Meier curves were used to assess processes that reached at least 10% improvement in a time-dependent manner. MedCalc® Statistical Software version 19.6 was used (MedCalc Software Ltd, Ostend, Belgium; https://www.medcalc.org; 2020).

## Results

The operating theatre of our institution in Bellinzona e Valli Regional Hospital consisted of more than one hundred professionals who worked in five operating rooms performing more than 7,000 surgical interventions annually and a circulating volume of surgical sets of 22,468 units in 2022. The composition of a surgical set varies depending on the intervention for which it is prepared, ranging from a single instrument to hundreds of instruments, with an average of 14 instruments per set. In 2022 a total number of 314,552 instruments passed through this supply chain.

**Figure 2:**
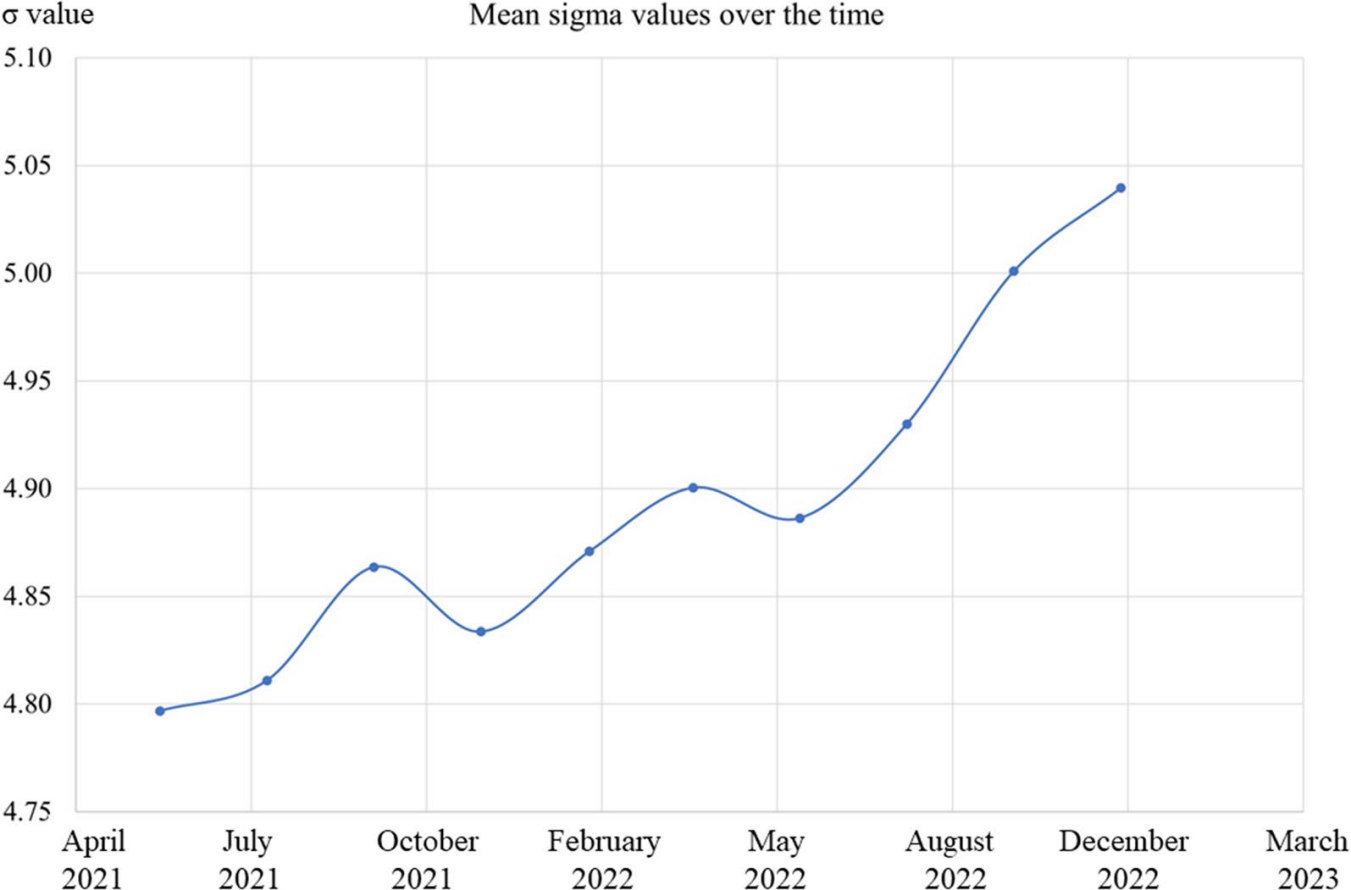
Sigma value improvement over the study period.

**Figure 3:**
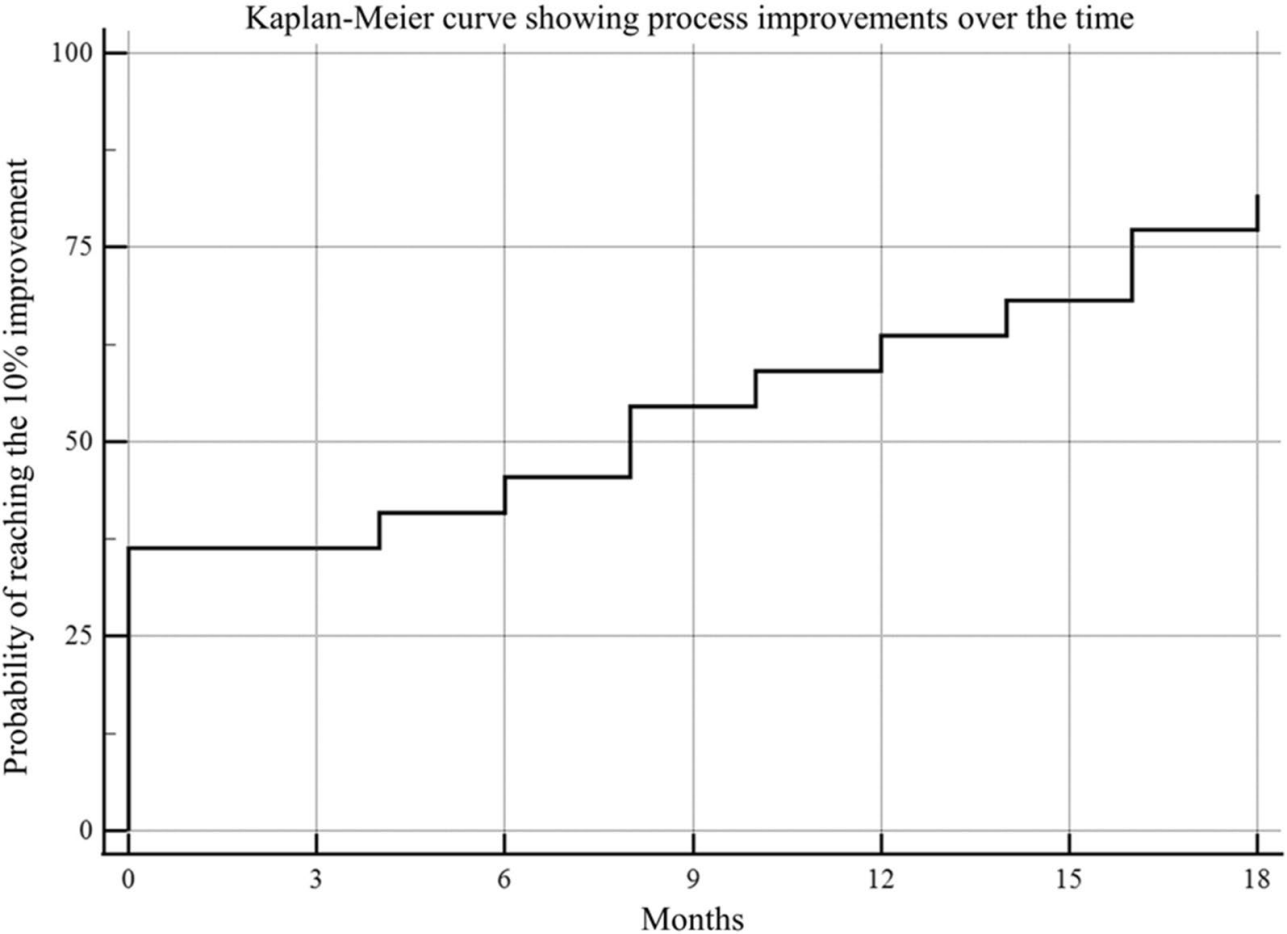
Percentage of processes reaching 10% sigma improvement during the study period.

During the study period of 18 months, twenty-two OR processes were assessed every 2 months. The initial mean value was 4.79 ± 1.02 σ and the final one was 5.04 ± 0.85 σ (SMD 0.60, 95%CI 0.16–1.04, p = 0.010). Figure 2. Intermediate values were: 4.81 ± 1.02 σ at 2 months, 4.86 ± 0.99 σ at 4 months, 4.83 ± 0.99 σ at 6 months, 4.87 ± 0.96 σ at 8 months, 4.90 ± 0.95 σ at 10 months, 4.89 ± 0.96 σ at 12 months, 4.93 ± 0.93 σ at 14 months, 5.00 ± 0.88 σ at 16 months, and 5.04 ± 0.85 σ at 18 months. This shows a continuous improvement trend over the 18 months of the project. In 8/22 (36%) processes the initial σ value was 6.00, while in 10/14 (71%) processes the threshold of 10% improvement of the initial σ value was reached (Fig. 3).

Such a significant improvement of 0.25 σ (+ 5.2%) was estimated to correspond to annualized 19,729 United States dollars (USD) of cost savings in the OR environment. Swiss franc (CHF) to the USD exchange rate: 1.09 (updated on January 26th, 2023).

Regarding personnel satisfaction, 150 questionnaires were answered, of which 84 were during the first 6 months and 66 were during the last 6 months. We received the questionnaire from 55 surgeons, 25 support equip members, and 70 OR nurses. The scores in the first and last 6 months were 7.3 ± 1.8 pts vs. 7.8 ± 1.5 pts for surgeons (p = 0.240), 7.1 ± 1.2 pts vs. 8.8 ± 0.7 pts for the support equipe (p = 0.005) and 7.3 ± 2.1 pts vs. 7.4 ± 1.5 pts for OR nurses. The overall score improved from 6.6 ± 2.2 pts to 7.0 ± 1.9 pts (0.013), with an increase of 6.0%. Subgroup analyses were conducted for each question and each group; however, no noteworthy finding was present (Table 3).

**Table 3:** Net promoter scores.

Questionnaire	Study begin	Study end	p
How likely would you recommend other colleagues to work with us?	7.3 (1.8)	7.8 (1.5)	0.061
How satisfied are you with the organization of the surgical instruments?	6.6 (1.6)	7.5 (1.3)	< 0.001
How often do defects occur in the instruments?	5.6 (2.2)	6.4 (2.1)	0.028
How often do you have to wait to schedule a surgery due to the lack of availability of the instruments?	6.4 (2.7)	6.6 (2.2)	0.049
How satisfied are you with the support provided by the instrument personnel during surgery?	6.6 (2.2)	6.4 (1.9)	0.169
How confident do you feel working safely with the instruments at your disposal?	7.1 (2.2)	7.6 (1.3)	0.017
How satisfied are you with the overall organization of the operating room?	7.0 (2.3)	7.3 (2.0)	0.536
Overall	6.6 (2.2)	7.0 (1.9)	0.002

Scores were presented as mean with standard deviation in parentheses.

## Discussion

In our study, we found out that the application of the Six Sigma lean methodology to the operating room context determined an improved efficiency and cost-effectiveness of the surgical instrument sterilization process, as well as an improvement in overall personnel satisfaction.

One key assumption of applying Six Sigma to healthcare sector is the availability of reliable and comprehensive data, a cornerstone of Six Sigma's data-driven approach. In contrast to manufacturing, healthcare data can be more complex due to diverse patient profiles and documentation variations. However, the intricate and human-centric nature of healthcare processes introduces complexities that can challenge this assumption^12,13^. The transition of Six Sigma to healthcare faces limitations stemming from the distinct attributes of the industry. The human-centric element, where clinical decisions intertwine with individual patient preferences, poses challenges. The application of Six Sigma's standardized procedures might not fully capture the nuances of personalized medical care. Moreover, healthcare's dynamic landscape marked by evolving medical knowledge and regulations creates a contrasting environment to the manufacturing sector's more stable processes^4^. Despite these challenges, Six Sigma exhibits promising potential applications in healthcare. For instance, the reduction of medication errors, similar to defect minimization in manufacturing, is achievable through targeted process improvements. The optimization of emergency room wait times and OR supply chain optimization draw parallels with reducing cycle times in manufacturing, demonstrating the adaptability of Six Sigma principles. Likewise, enhancing surgical procedures mirrors the refinement of industrial processes. In practice, a hospital successfully employed Six Sigma to enhance surgical processes, leading to improved patient outcomes. While assumptions and limitations must be acknowledged, the integration of Six Sigma into healthcare holds significant promise. It provides a structured framework for process enhancement and quality improvement. By recognizing the nuances of healthcare's human-centered and dynamic nature, Six Sigma can be effectively tailored to drive positive outcomes and efficiencies in healthcare delivery^15^.

We found that our results were in line with findings from other published studies. For instance, Egan et al.^22^ conducted a study that focused on surgical process improvement using Six Sigma principles. They reported a 55% decrease in overall nursing time spent in gathering and preparing materials for surgical cases, with a corresponding reduction in packaging waste. This enabled nurses to focus on continuing to deliver high-quality care, and improved overall efficiency. Furthermore, a study by Schön et al.^23^ examined the impact of Six Sigma implementation on employee morale and satisfaction in a hospital setting. They observed a marked increase in job satisfaction among healthcare staff due to improved communication, reduced process variability, and enhanced overall operational efficiency. This mirrors our study's findings of improved personnel satisfaction as a direct result of Six Sigma application. Similar results were also achieved in the study of Godley et al.^16^. Ultimately, the study carried out by O'Mahony et al.^24^ employed Six Sigma methodology to streamline the operating room's supply chain. The study's authors identified an overall decrease of 17.7% in the value of stock inventory within the operating theater, an impressive reduction of 91.7% in stock items reaching their expiration, and a remarkable 45% reduction in clinical staff time spent preparing necessary stock for procedures. These outcomes effectively underscore the efficiency of Lean Six Sigma in managing healthcare supply chains. These conclusions align closely with our study's own findings of cost-saving advantages stemming from the optimization of operating room processes.

In our own experience, we've encountered the same limitations and challenges when applying Six Sigma methodologies. The assumptions we made about data availability and process standardization were tested as we delved into the complexities of healthcare processes. Despite our best efforts, obtaining consistent and accurate data proved to be a significant hurdle due to the inherent variability in patient information and documentation practices. Moreover, the human-centric nature of healthcare presented challenges similar to those seen in the industrial sector. Just as Six Sigma may not fully capture the intricacies of patient preferences and clinical judgment, we faced instances where standardized approaches struggled to account for the nuanced decision-making required in patient care. This highlighted the need for a more tailored approach that respects the human elements involved. These variables introduced a level of uncertainty that necessitated ongoing adjustments to our strategies.

At our institution, the Six Sigma methodology was implemented as an operating room management tool, in sequential steps over the course of a few months. These steps are summarized by the acronym DMAIC: Define (issues identification), Measure (collecting data), Analyze (statistical analysis of data collected), Improve (introduce corrections), Control (monitor data trends to assess the impact of corrections)^17,25,26^. Those steps are not per se innovative, as they essentially retrace, with further elaboration, the quintessential Deming’s cycle (Plan-Do-Check-Act), which is considered a milestone in the culture of continuous improvement and quality management systems, today deeply embedded into the modern managerial culture. The peculiarity of Six Sigma, however, consists in the systematic use of statistics, aimed at analyzing and continuously shrinking the variability of a given process concerning the gold standard, once this standard has been identified as the best possible^4^.

While these limitations were encountered, they also provided valuable insights. They reinforced the importance of adapting Six Sigma principles to the unique realities of healthcare^27^. Our experience underscored the need for flexibility, a deeper understanding of patient-centric factors, and an ongoing commitment to refining processes in response to changing dynamics. This aligns with the broader notion that Six Sigma's successful integration into healthcare demands a nuanced approach that acknowledges and navigates these challenges. In our experience, understanding and applying the Lean Six Sigma methodology required time and work at the very beginning. It was key to have a designated project leader within the OR team, responsible for its implementation and relying on a Lean Six Sigma Master Black Belt external advisor. However, after 1 year the return on investment proved worthwhile, with a clear saving in terms of COPQ. Moreover, beyond a merely economical point of view, the application of Six Sigma did increase the overall satisfaction in both the OR and the surgical team, measured in terms of NPS. The NPS is also a methodology born in the industrial world in 2003. It has been used in various fields, including banking and insurance. In recent years, it has found application in the healthcare industry as well, as a parameter for objectifying the degree of satisfaction of both internal and external stakeholders and patients, and proved itself very useful for benchmarking analysis. This type of evaluation scale is nowadays widely used in the healthcare context^14^.

This study has some limitations. Although the implementation of the Lean Six Sigma methodology was prospective, the analysis of data was retrospective. Cost saving was deducted with a top-down approach, calculating the COPQ associated with the one decimal point of the sigma value and assessing the reduction in the latter. The applicability and generalizability of our results may be limited as each operating suite has its dynamic, organization, and processes. Another limitation is related to the statistical analysis. In our study, uncertainty and heterogeneity of collected data were consistent and may be handled in future studies with neutrosophic statistics, an extension of classical statistics which is applied when the data is coming from a complex process or from an uncertain environment^28–30^. Further studies in different institutions and operating suites are needed to better assess the actual impact of the application of the Lean Six Sigma methodology on operating room economics.

In our experience the application of the Lean Six Sigma methodology to the process of surgical instruments sterilization was cost-effective, significantly decreasing the operating suits COPQ and increasing internal stakeholder overall satisfaction.

## Data Availability

The dataset analysed during the current study is available from the corresponding author on request.
